# Functional expression of the mechanosensitive PIEZO1 channel in primary endometrial epithelial cells and endometrial organoids

**DOI:** 10.1038/s41598-018-38376-8

**Published:** 2019-02-11

**Authors:** Aurélie Hennes, Katharina Held, Matteo Boretto, Katrien De Clercq, Charlotte Van den Eynde, Arne Vanhie, Nele Van Ranst, Melissa Benoit, Catherine Luyten, Karen Peeraer, Carla Tomassetti, Christel Meuleman, Thomas Voets, Hugo Vankelecom, Joris Vriens

**Affiliations:** 1 Laboratory of Endometrium, Endometriosis & Reproductive Medicine, Department of Development and Regeneration, KU Leuven, Herestraat 49 box 611, 3000 Leuven, Belgium; 2 Laboratory of Ion Channel Research, Department of Cellular and Molecular Medicine, KU Leuven, VIB Center for Brain & Disease Research, Herestraat 49 box 802, 3000 Leuven, Belgium; 3 Laboratory of Tissue Plasticity in Health and Disease, Cluster of Stem Cell and Developmental Biology, Department of Development and Regeneration, KU Leuven, Herestraat 49 box 804, 3000 Leuven, Belgium; 4 Leuven University Fertility Centre, University Hospitals Leuven, Herestraat 49, 3000 Leuven, Belgium

## Abstract

Successful pregnancy requires the establishment of a complex dialogue between the implanting embryo and the endometrium. Knowledge regarding molecular candidates involved in this early communication process is inadequate due to limited access to primary human endometrial epithelial cells (EEC). Since pseudo-pregnancy in rodents can be induced by mechanical scratching of an appropriately primed uterus, this study aimed to investigate the expression of mechanosensitive ion channels in EEC. Poking of EEC provoked a robust calcium influx and induced an increase in current densities, which could be blocked by an inhibitor of mechanosensitive ion channels. Interestingly, RNA expression studies showed high expression of *PIEZO1* in EEC of mouse and human. Additional analysis provided further evidence for the functional expression of PIEZO1 since stimulation with Yoda1, a chemical agonist of PIEZO1, induced increases in intracellular calcium concentrations and current densities in EEC. Moreover, the ion channel profile of human endometrial organoids (EMO) was validated as a representative model for endometrial epithelial cells. Mechanical and chemical stimulation of EMO induced strong calcium responses supporting the hypothesis of mechanosensitive ion channel expression in endometrial epithelial cells. In conclusion, EEC and EMO functionally express the mechanosensitive PIEZO1 channel that could act as a potential target for the development of novel treatments to further improve successful implantation processes.

## Introduction

Embryo implantation is a fundamental step in reproduction that requires an intimate interaction between a competent blastocyst and a receptive endometrium^1,2^. Active embryo selection at the site of implantation requires the appropriate embryonic signals to be perceived and translated by the endometrium^3^. The current insights into the molecular mechanisms in which chemical and/or physical signals released by the blastocyst and detected by the endometrial epithelial cells (EEC), are still obscure. Ultrastructural animal studies of early stages of implantation have demonstrated a physical interaction between the embryo and the endometrial epithelium^4^. Decidualization, known as the progesterone-dependent differentiation of fibroblast-like endometrial stromal cells into large, secreting decidual cells, is a key step to achieve successful implantation. Interestingly, the decidualization reaction in rodents can be induced in the absence of an embryo by the application of physical signals such as intraluminal injection of oil, or scratching of the endometrium^5^. The signaling role of the endometrial epithelium in processing these physical signals is indispensable since physically stimulated decidualization does not take place when the epithelium is destroyed or removed^6^. In humans, decidualization occurs spontaneously during the luteal phase of the menstrual cycle, in the absence of a blastocyst. However, clinical studies in women with previous repeated *In Vitro* Fertilization (IVF) failure suggest that endometrial injury, before IVF treatment, is associated with increased rates of implantation^7–9^. Nevertheless, the molecular mechanism behind this phenomenon and the involvement of mechanosensitive molecules are yet to be unraveled. Mechanosensitive ion channels are attractive candidates as transducers to transform the physical stimulus into an electrical signal. Earlier studies have reported the epithelial sodium channel (ENaC), a proposed mechanosensor^10,11^, as a regulator of the prostaglandin E_2_ production by the endometrial epithelium, a molecule that is required for embryo implantation^12^. Interestingly, several other ion channels, including the family of PIEZO channels^13^, and the polymodal members of the Transient Receptor Potential (TRP) superfamily, have been described as mechanosensitive^14–23^. PIEZO1 expression is described in lungs, bladder, pancreas and skin, where mechanosensation has important biological roles. However, unlike PIEZO2, which is highly expressed in sensory dorsal root ganglia, PIEZO1 is not expressed in sensory neurons^13^. This study aims to provide evidence for the endogenous expression of mechanosensitive ion channels in EEC of human and mouse.

Ethical and practical considerations often limit the use of primary human endometrial epithelial cells (hEEC) for research purposes. Even more, hEEC have proven difficult to isolate and to culture, resulting in the use of endometrial epithelial cancer cell lines for research. However, their physiological relevance as a model for endometrial epithelial cell can be questioned^24^. Recently, 3D human endometrial organoids (EMO) were demonstrated to represent a valuable model for hEEC, reproducing phenotypical and physiological aspects of the tissue, and can provide an important tool to study the different aspects of implantation^25^. Moreover, the organoids are long-term expandable while retaining their properties, thereby providing a more accessible source of endometrial epithelial cells. Here, we evaluate the potential of EMO as a valid model for primary human EEC to investigate the embryo-uterine crosstalk by studying the functional expression of mechanosensitive ion channels.

## Results

### Mechanosensitivity in human endometrial epithelial cells

**Figure 1:**
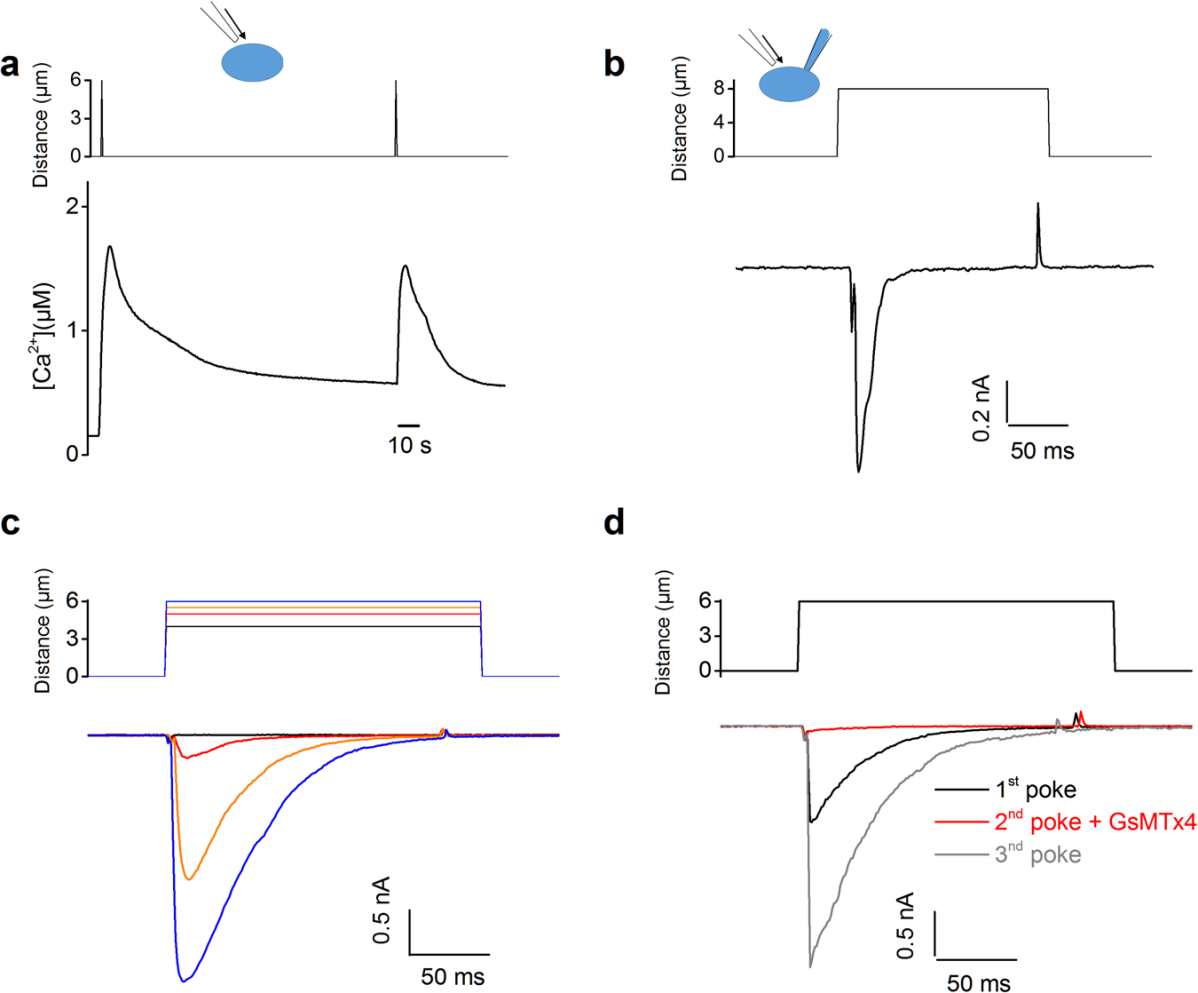
Functional expression of a mechanically activated ion channel in primary human EEC. **(a)** Representative trace of ratiometric Ca^2+^ imaging (Fura2) on human EEC. Cells were subjected to a series of mechanical stimuli by pressing a glass probe onto the cell surface for 100 ms (n = 10). **(b**–**d)** Mechanically activated currents of hEEC recorded in the whole-cell configuration. **(b)** Representative trace of mechanically activated inward current at −60 mV in hEEC (n = 4). **(c)** Representative currents subjected to a series of mechanical steps in 1 µm increments at −60 mV in hEEC. **(d)** Representative traces of mechanically activated currents evoked at −60 mV in the presence of GsMTx4 (20 µM).

Primary cultures of human EEC (hEEC) were established starting from endometrial biopsies. The matrix-metalloproteinase 2 and 7 (MMP-2 and MMP-7) were used as markers to confirm the epithelial character of the endometrial cells^26^. Typically, hEEC showed low mRNA expression of the stromal marker *MMP-2*, whereas the epithelial marker *MMP-7* was highly expressed. In addition, these results were in line with the positive immunostaining for MMP-7 (Supplementary Fig. S1). Interestingly, mechanical stimulation of hEEC, by poking of the cell membrane, induced a robust and transient Ca^2+^ influx (mean ΔCa^2+^  = 1315 ± 335 nM) (Fig. 1a). When Ca^2+^ was omitted from the extracellular medium similar mechanical stimulation of the plasma membrane did not induce any increase in intracellular Ca^2+^ concentration. However, application of ionomycin after poking of the cells evoked an increase in intracellular Ca^2+^ concentration by depletion of the Ca^2+^ stores (Supplementary Fig. S2). Further validation of the functional expression of mechanosensitive ion channels was performed using the whole-cell patch clamp technique in order to measure direct channel activation. Mechanical poking of hEEC induced transient increases in current densities (Fig. 1b) which were rapid (latencies < 1 ms), and voltage dependent (mean current increase = 572 ± 22 pA and −915 ± 22 pA at +50 mV and −60 mV respectively) and showed a time dependent activation (τ ~ 1.14 ± 0.43 ms at −60 mV) and inactivation (τ ~ 22.4 ± 2.9 ms at −60 mV). The increase in current density by application of a mechanical stimulus was dependent of the depth of the poking since increased poking distances enlarged the current amplitude (Fig. 1c). Note that a minimal indentation of 5 µm was required to induce a current increase (Fig. 1c). The mechanosensitivity of hEEC was fully inhibited in the presence of GsMTx4, a peptide widely used to block mechanically activated channels^27^ (Fig. 1d). Altogether, these results demonstrated the endogenous expression of a mechanosensitive ion channel in hEEC.

### The mechanosensitive PIEZO1 channel is functionally present in human EEC

**Figure 2:**
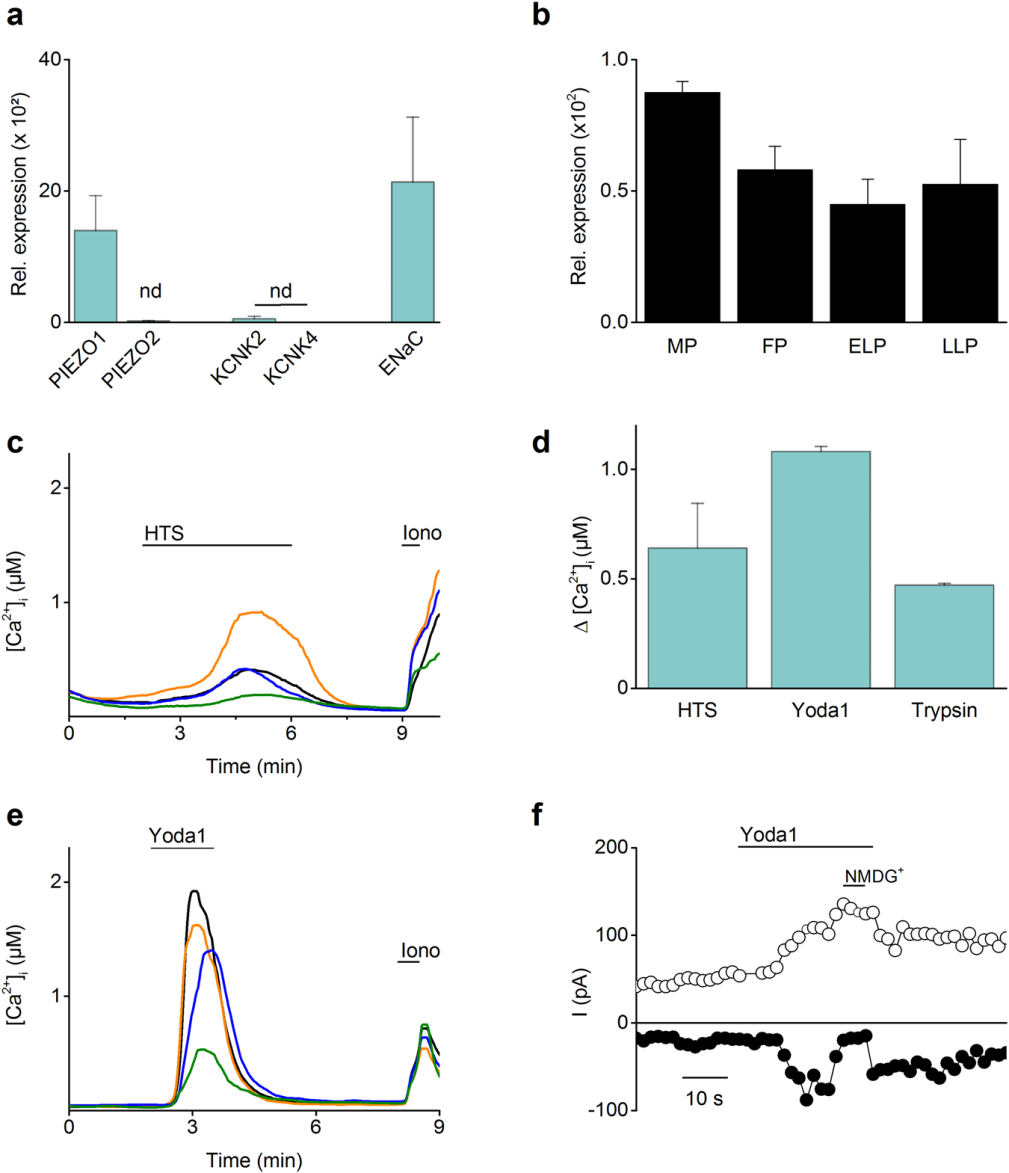
Functionality of the mechanosensitive PIEZO1 channel in hEEC. (**a**) mRNA expression levels of mechanosensitive ion channels in primary hEEC. mRNA levels were relatively quantified to the geometric mean of the housekeeping genes *HPRT1* and *PGK1* and represented as mean ± SEM. n = 4, Nd = not detectable. (**b**) RT-qPCR results of *PIEZO1* mRNA expression levels in human biopsies taken at different stages of the menstrual cycle: menstrual phase (MP, days 1–5, n = 6), follicular phase (FP, days 6–14, n = 21), early luteal phase (ELP, days 15–20, n = 15) and late luteal phase (LLP, days 21–28, n = 9). (**c**–**e**) Time course of Ca^2+^ experiments in which a hypotonic solution (HTS; 210 mOsm) (**c**) or the PIEZO1 agonist (**e**) was added to hEEC. Shown are four representative traces, at the indicated time points HTS, Yoda1 (5 µM) or ionomycin (Iono, 2 µM) were added to the cells. n = 3 independent experiments, with a total of minimal 350 cells. Ionomycin was applied at the end of each experiment as a positive control. (**d**) Mean Ca^2+^ increase of responding cells to HTS, Yoda1 (5 µM) or trypsin (2 µg/ml). Amplitudes are represented as mean ± SEM. (**f**) Time course at ±80 mV of a whole-cell patch clamp recording showing the effect of Yoda1 (10 µM) in primary hEEC (n = 4). At the indicated period, Na^+^ and Ca^2+^ were replaced by NMDG^+^.

To characterize the molecular identity of the mechanosensitive ion channels in hEEC, the expression of candidate mechanosensitive ion channels, including members of the PIEZO family^13^ and the two-pore K^+^ (KCNK) family^28,29^, were examined. RT-qPCR experiments showed detectable mRNA expression levels of *PIEZO1* (Ct < 34), while the expression levels of *PIEZO2*, *KCNK2* and *KCNK4* were below the detection level (Ct > 34). As a control, high levels of mRNA expression were detected for the *ENaC* channel, supporting the epithelial character of the cells (Fig. 2a). In addition, gene expression of *PIEZO1* was also detected in human endometrial stromal cells (Supplementary Fig. S3) and in human endometrial biopsies (Fig. 2b). Induced cell swelling via application of a hypotonic solution (HTS) resulted in a Ca^2+^ influx in hEEC (mean ΔCa^2+^  = 640 ± 204 nM) (Fig. 2c,d). Furthermore, application of the selective chemical agonist of the PIEZO1 channel, Yoda1^30^, induced robust increases in intracellular Ca^2+^ concentration (mean ΔCa^2+^  = 1081 ± 23 nM) (Fig. 2d,e). In whole-cell patch clamp experiments, application of Yoda1 evoked an increase in both in- and outwardly rectifying current densities (mean ΔI = −97.9 ± 50.5 pA and 69.8 ± 29.7 pA at −80 mV and +80 mV respectively) with an average reversal potential of +2.3 mV (Fig. 2f). To confirm the epithelial character of the cells, functional expression of ENaC was evaluated via stimulation with trypsin, a protease activator of the channel^31^, which resulted in a Ca^2+^ influx (mean ΔCa^2+^  = 470 ± 8 nM) (Fig. 2d) in 98.5% of the cells. Together, these results provide evidence for the endogenous expression of the mechanosensitive PIEZO1 in hEEC.

### Functional expression of mechanosensitive ion channels in mouse EEC

**Figure 3:**
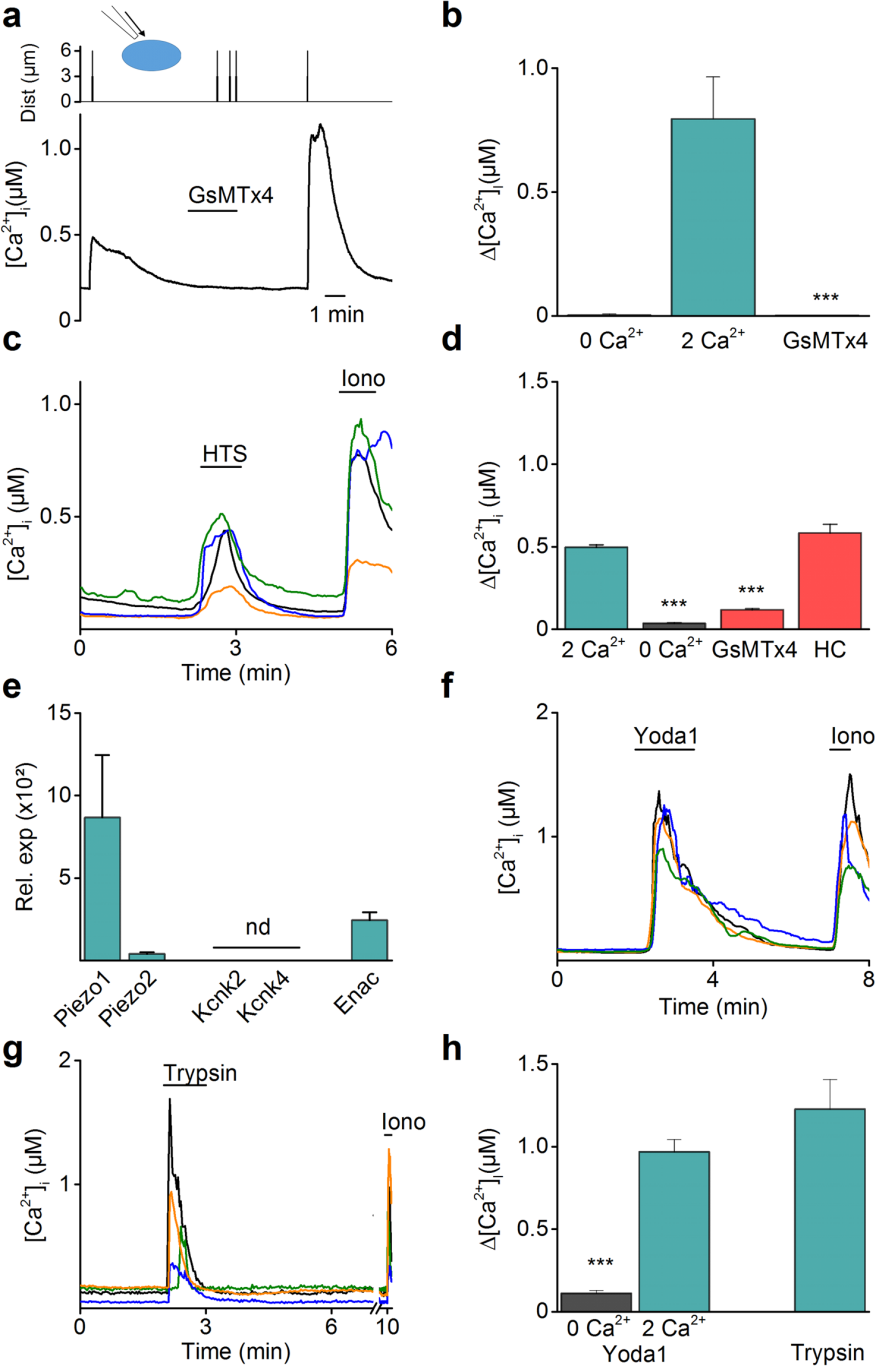
Mechanosensitive ion channels in primary EEC of mouse. (**a**) Time course showing the intracellular Ca^2+^ concentration of mouse EEC upon mechanical stimulation (5 µm for 100 ms) at the indicated time points. When indicated the spider toxin GsMTx4 (20 µM) was added to the external solution (n = 12). (**b**) Mean amplitude of [Ca^2+^]_I_ in mEEC, represented as the difference between the peak value and the baseline value after mechanical poking of the cells in 0 mM extracellular Ca^2+^, 2 mM Ca^2+^ and in the presence of the inhibitor GsMTx4 (20 µM). Data are represented as mean ± SEM. ***p < 0.001 using the non-parametric Kruskal-Wallis test compared to 2 mM Ca^2+^ conditions. (**c**) Example traces of hypotonic (HTS), (210 mOsm) – induced intracellular Ca^2+^ change in mEEC. N = 3 independent experiments with a total minimum of 1500 cells. (**d**) Mean amplitude of [Ca^2+^]_I_ in mEEC, after application of HTS in an extracellular solution containing 0 mM, 2 mM Ca^2+^ and in the presence of GsMTx4 (20 µM) or the TRPV4 inhibitor HC-067047 (HC; 100 nM). ***p < 0.001 using the non-parametric Kruskal-Wallis test corrected with Dunn’s multiple comparisons to 2 mM Ca^2+^ conditions. N = 3 independent experiments with a total minimum of 800 cells. (**e**) mRNA expression levels of mechanosensitive ion channels in primary mEEC. mRNA levels were relatively quantified to the geometric mean of the housekeeping genes *Tbp* and *Pgk1* and represented as mean ± SEM (n = 3). Nd = not detectable. (**f**,**g**) Depict stimulation of cells with either Yoda1 (5 µM) or trypsin (20 µg/ml) to induce intracellular Ca^2+^ influxes. Ionomycin (Iono, 2 µM) was applied at the end of each experiment as a positive control. Each line represents a single cell and four representative traces are shown. n = 3 experiments, with a total of minimum 180 cells per condition. (**h**) Mean Ca^2+^ amplitude of responding cells to Yoda1 (5 µM) and trypsin (20 µg/ml) in normal medium containing 2 mM external Ca^2+^ and Ca^2+^ free conditions. Amplitudes are represented as mean ± SEM. ***p < 0.001 using the non-parametric Mann-Whitney U test compared to 2 mM Ca^2+^ conditions.

Similarly, the expression of candidate mechanosensitive ion channels was investigated in primary mouse endometrial epithelial cells (mEEC)^32^. The epithelial character of mEEC was validated by immunostaining for the markers MMP-2 and MMP-7^26^ (Supplementary Fig. S4). Next, mechanical poking of mEEC resulted in a rapid and reversible Ca^2+^ influx (mean ΔCa^2+^  = 795 ± 169 nM) (Fig. 3a,b). However, when Ca^2+^ was omitted from the extracellular medium, mechanical stimulation of the cell membrane did not induce any Ca^2+^ influx (mean ΔCa^2+^  = 2 ± 4 nM) (Fig. 3b). Similar to hEEC, incubation by GsMTx4 induced a full block (100%) of the mechanically evoked Ca^2+^ influx (Fig. 3a,b). Moreover, application of a hypotonic solution (HTS) resulted in an increase in Ca^2+^ concentration (mean ΔCa^2+^  = 496 ± 15 nM) (Fig. 3c) which was strongly reduced in the absence of extracellular Ca^2+^ (mean ΔCa^2+^  = 36 ± 3 nM) in mEEC. The HTS-induced Ca^2+^ influx was significantly blocked in the presence of GsMTx4 (mean ΔCa^2+^  = 118 ± 6 nM) while incubation with the TRPV4 antagonist HC-067047^33^ (HC) was without effect on the HTS-induced Ca^2+^ influx (mean ΔCa^2+^  = 583 ± 53 nM) (Fig. 3d).

RT-qPCR experiments on mEEC displayed detectable mRNA expression of *Piezo1* and *Enac* channels (Ct < 34), while no gene expression was detected for *Piezo2*, *Kcnk2* and *Kcnk4* (Ct > 34) (Fig. 3e). Detectable mRNA levels for *Piezo1* were also observed in mouse endometrial stromal cells (mESC) (Supplementary Fig. S3). Moreover, poking of the plasma membrane of mouse PIEZO1 (mPIEZO1) overexpressing HEK-293T cells induced a transient Ca^2+^ influx (mean ΔCa^2+^  = 918 ± 109 nM) which was comparable to the Ca^2+^ influx in mEEC after poking of the cell (Supplementary Fig. S5). In contrast, application of the poking protocol to non-transfected HEK-293T cells did not induce any increase in intracellular Ca^2+^ (mean ΔCa^2+^  = 7 ± 2 nM). PIEZO1 overexpressing cells showed an increase in current density after poking of the cell membrane in whole cell patch clamp experiments, exhibiting a similar time dependent activation and inactivation as in hEEC^34^ (τ ~ 2.34 ms and τ ~ 8.67 ms for activation and inactivation respectively) (Supplementary Fig. S5). In addition, stimulation of mEEC by Yoda1 produced a reversible rise in intracellular Ca^2+^ (mean ΔCa^2+^  = 968 ± 75 nM) (Fig. 3f,h). The percentage of Yoda1 responders and the amplitude of the Ca^2+^ influx were significantly reduced when Ca^2+^ was omitted from the extracellular solution (only 4.8% responders, mean ΔCa^2+^  = 110 ± 17 nM) (Fig. 3h). Similar results were observed in HEK-293T cells overexpressing mPIEZO1, which showed strong Ca^2+^ increases after Yoda1-stimulation (mean ΔCa^2+^  = 570 ± 56 nM) whereas only 2.5% of non-transfected cells showed responses to Yoda1, with significantly reduced Ca^2+^ influx (p < 0.001) (mean ΔCa^2+^  = 132 ± 6 nM) compared to mPIEZO1 transfected cells (Supplementary Fig. S5). Furthermore, stimulation of mEEC with trypsin induced a strong Ca^2+^ influx (ΔCa^2+^  = 1227 ± 179 nM) in 56.7% of the cells (Fig. 3g,h), which was comparable to earlier reports showing the trypsin-induced calcium influxes in mouse endometrial cells^12^ and validates the epithelial character of the investigated cells.

**Figure 4:**
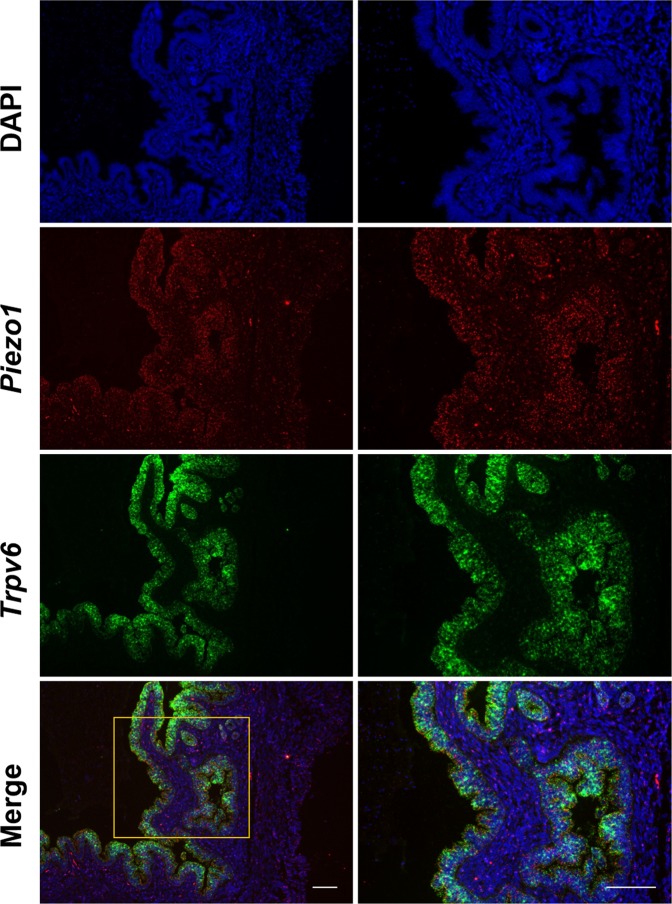
*In Situ* hybridization showing *Piezo1* mRNA expression in endometrial epithelial cells. *In situ* hybridization (ISH) on paraffin section from the uterine horn. The bottom panel shows the merged image obtained from DAPI (blue), *Piezo1* (red) and *Trpv6* (green color) stainings. Right panels are magnification of a specific region shown in the left image. Pictures were taken using a 10x objective. Scale bar: 100 µm.

Interestingly, *in situ* hybridisation (RNAscope) for *Piezo1* in sections of the uterine horn revealed a prominent signal for *Piezo1* RNA in luminal epithelial cells, while the fluorescent signal intensity in the stromal part was lower (Fig. 4). The specificity of the RNA probes was evaluated in bladder urothelium^35^ and trigeminal neurons^13^ as positive and negative control tissue, respectively (Supplementary Fig. S6). To validate the expression of *Piezo1* RNA in the epithelial cells, *in situ* hybridisation for *Trpv6* was tested, showing positive *Trpv6* signals, exclusively in the luminal and glandular epithelial cells of the uterine horn. These results are in line with earlier reports showing typical TRPV6 expression in the endometrial epithelial cells of the mouse^36^. Merging both *Piezo1* and *Trpv6* signals, resulted in a positive staining for both channels in the endometrial epithelial cells. Taken together, these experiments provide evidence for the expression of PIEZO1 in endometrial epithelial cells of the mouse.

### TRP channels in human EEC

**Figure 5:**
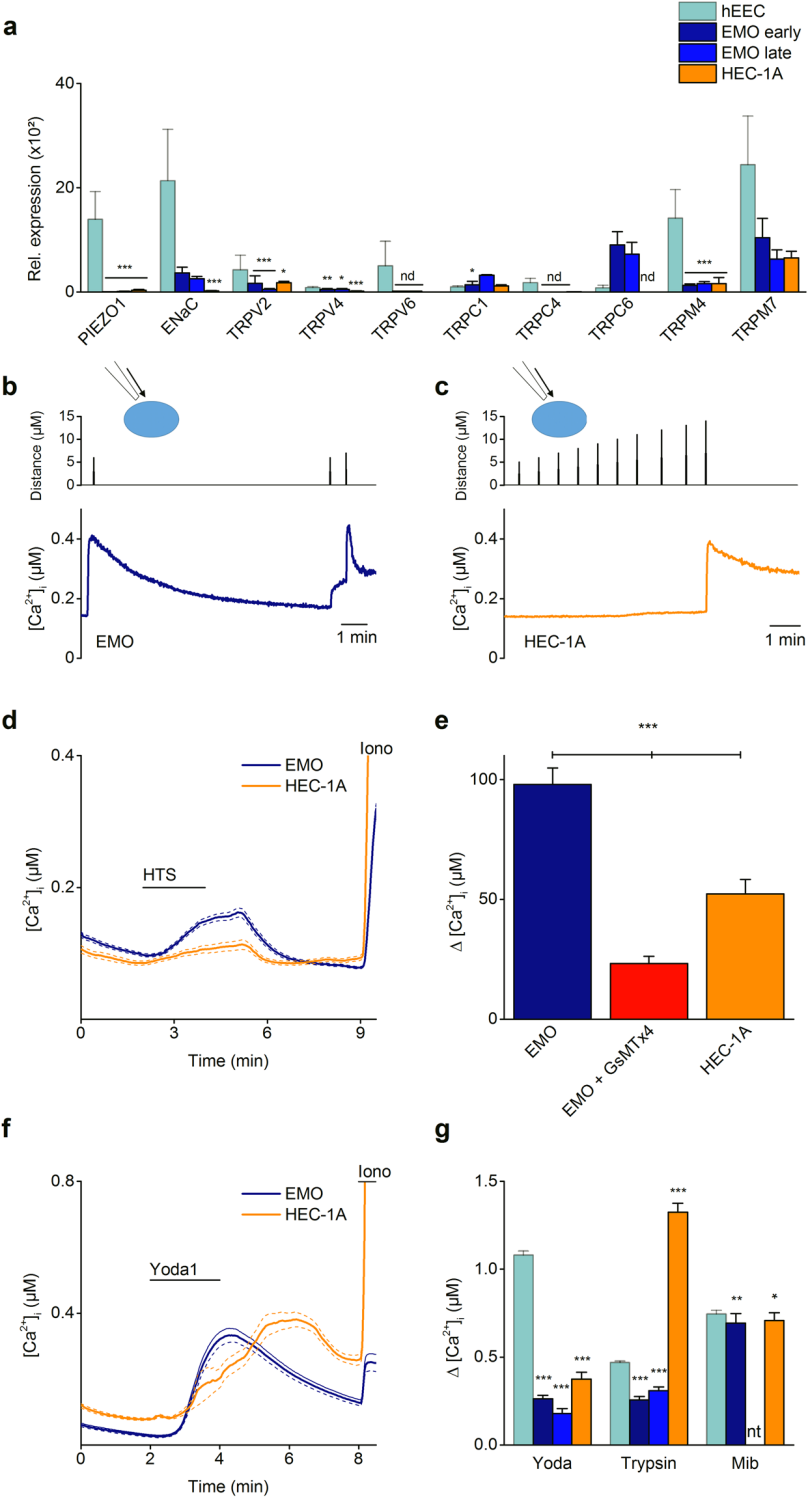
EMO as a model for human endometrial epithelial cells. (**a**) mRNA expression levels of selected ion channels in primary hEEC (light blue), early (P2) (blue) and late (P8) (dark blue) passage EMO, and the endometrial epithelial cell line HEC-1A (orange). mRNA levels were relatively quantified to the geometric mean of the housekeeping genes *HPRT1* and *PGK1* and represented as mean ± SEM. n = 4 (hEEC), n = 3 (EMO). *p < 0.05, **p < 0.01 compared to the hEEC condition with Two-way Anova and Dunnett’s multiple comparison test; Nd = not detectable. (**b**–**g**) Ca^2+^ microfluorimetry. Representative trace of ratiometric Ca^2+^ imaging (Fura2) on EMO (**b**) and HEC-1A (**c**). Cells were subjected to a series of mechanical stimuli by pressing a glass probe onto the cell surface for 100 ms (N ≥ 10 independent experiments). (**d**) Time course of Ca^2+^ experiments in which a hypotonic solution (HTS; 210 mOSm) was added to either EMO or HEC-1A cells at the indicated time point. Ionomycin (Iono; 2 µM) was applied as a positive control. Shown is the mean trace ± SEM. The corresponding mean amplitude of [Ca^2+^]_I_ upon application of HTS in either 2 mM extracellular Ca^2+^ or in the presence of GsMTx4 (20 µM) is shown in (**e**). ***p < 0.001 using the non-parametric Kruskal-Wallis test corrected with Dunn’s multiple comparisons. n = 3 experiments with a total minimum of 200 cells. (**f**) Time course of intracellular Ca^2+^ in EMO and HEC-1A upon stimulation with Yoda1 (5 µM). Ionomycin (Iono; 2 µM) was applied as a positive control. Shown is the mean trace ± SEM. (**g**) represents the mean amplitude of [Ca^2+^]_I_ upon application of Yoda1, trypsin or mibefradil (mib) in either hEEC, early passage EMO, late passage EMO or HEC-1A cells. *p < 0.05, **p < 0.01, ***p < 0.001 using the non-parametric Kruskal-Wallis test corrected with Dunn’s multiple comparisons compared to the hEEC conditions. n = 3 experiments with a total minimum of 80 cells. nt = not tested.

In earlier reports, several polymodal members of the TRP superfamily were proposed as candidates involved in sensing mechanical forces^37^. Therefore, hEEC were evaluated for the gene expression pattern of a selected number of TRP channels based on earlier expression studies performed on human endometrial biopsies^38^. RT-qPCR experiments showed detectable mRNA expression levels of *TRPV1*, *TRPV2*, *TRPV4*, *TRPV6*, *TRPC1*, *TRPC4*, *TRPC6*, *TRPM4* and *TRPM7* (Ct values < 34), with *TRPV6*, *TRPM4* and *TRPM7* showing the highest expression levels. In contrast, expression levels for *TRPA1*, *TRPC3*, *TRPC5*, *TRPC7*, *TRPM1*, *TRPM2*, *TRPM3*, *TRPM5*, *TRPM6* and *TRPM8* were below detection limit (Ct values ≥ 34) (Supplementary Fig. 7). Next, the functional expression of these TRP channels was tested using Ca^2+^ microfluorimetry experiments (Supplementary Fig. 7). As specific TRP channel pharmacology is scarce, only a limited number of TRP channels could be tested. Cells were stimulated with *Δ*^9^*-*tetrahydrocannabinol (THC), GSK1016790A (GSK), 1-oleoyl-2-acetyl-glycerol (OAG), (−) Englerin A (EA) or mibefradil (Mib) to assess the functionality of TRPV2, TRPV4, TRPC6, TRPC1/C4 heteromultimers, or TRPM7 respectively. A significant calcium response was observed upon stimulation of hEEC with the TRPM7 agonist mibefradil^39^ (mean ΔCa^2+^  = 1038 ± 58 nM) (Fig. 5g and Supplementary Fig. 7). However, none of the other TRP channel agonists induced a significant increase in calcium concentration in hEEC (% of responding cells ≤ 2). Altogether, these results showed functional expression of TRPM7, while no functional expression was detected for TRPV2/V4 and TRPC1/C4/C6 in human endometrial epithelial cells, excluding the potential contribution of polymodal TRP channels in the mechanosensitivity of hEEC.

### Endometrial organoids as a model to investigate ion channel activity

Acquiring sufficient material for the culture of human endometrial epithelial cells is often bound to ethical and practical issues, and provides a challenge in terms of long-term culture. Popular endometrial epithelial cell lines are derived from cancers and do not fully mimic the cellular background of the inner layer of the uterine wall. Recently, a new tool emerged, i.e. endometrial epithelium organoids^25,40^. As organoid epithelial cells are derived from identical starting material as primary hEEC, their physiology may be more closely related to primary cells as compared to the available endometrial epithelial cancer cell lines. To validate whether endometrial organoids relate to primary hEEC in terms of ion channel functionality, the expression pattern of several channels was investigated. To start, organoids were dissociated into single cells and seeded as a monolayer, thereby revealing a morphology comparable to hEEC. After validating the epithelial character of EMO-derived cells (Supplementary Fig. S8), the expression of mechanosensitive ion channels and TRP channels was evaluated in early passage (P2) intact endometrial organoids (EMO). Surprisingly, gene expression of *PIEZO1* was undetected (Ct > 35), whereas gene expression of *ENaC* was observed (Ct < 34) in the intact EMO, albeit at a significantly lower level (Fig. 5a). However, RT-qPCR results from 2D and 3D cultures of organoids showed increased *PIEZO1* RNA expression upon 2D culture conditions (Supplementary Fig. S9), indicating potential alterations in gene expression induced by differences in culture conditions. In addition, the expression pattern of TRP channels in EMO was similar compared to hEEC (Ct < 34), although most levels of expression tended to be lower. Nevertheless, a positive correlation was shown for the TRP channel expression levels comparing hEEC and EMO (Spearman correlation = 0.66; p < 0.05) (Supplementary Fig. 7).

As long-term expandability represents an important asset of organoids, expression of a selected panel of ion channels was tested after long-term culture (Passage (P) 8). RT-qPCR analysis showed a steady expression profile for all of the examined channels as compared to early (P2) passage EMO (Fig. 5a).

Mechanical stimulation of 2D cultures of EMO induced Ca^2+^ influxes after poking of the cell membrane or application of a hypotonic solution (mean ΔCa^2+^  = 98 ± 7 nM). The HTS-induced increase in intracellular Ca^2+^ was significantly reduced in the presence of GsMTx4 (Fig. 5b,d,e). Moreover, ligand activation of PIEZO1 evoked a Ca^2+^ influx in early and late passage (P8) EMO (mean ΔCa^2+^  = 262 ± 19 nM and 179 ± 28 nM for P2 and P8 respectively). However, the mean amplitude of the Ca^2+^ influx after mechanical and chemical stimulation of EMO was lower as compared to hEEC (Fig. 5g). Stimulation of EMO derived cells with TRP channel agonists (EA, GSK, OAG and THC) did not induced a significant calcium influx, while robust responses were detected after application of mibefradil that were somewhat reduced compared to primary hEEC (mean ΔCa^2+^  = 745 ± 21 nM vs 694 ± 54 nM for hEEC and EMO respectively) (Fig. 5g).

To validate EMO as model for endometrial epithelial cells, analogue experiments were performed on the endometrial cancer cell line HEC-1A^41^. Overall RT-qPCR experiments showed significantly low(-er) gene expression levels in HEC-1A cells for some of the assessed ion channels compared to hEEC (Fig. 5a). Correlation studies showed distinct ion channel expression patterns for hEEC and HEC-1A (Spearman correlation = 0.52; p = 0.13) (Supplementary Fig. S10). At the functional level, HEC-1A cells showed calcium responses to mechanical poking of the cell membrane and to application of a hypotonic solution. However, the sensitivity to mechanical stimulation was remarkably lower in HEC-1A cells as the minimal indentation to induce a Ca^2+^ response (mean minimal indentation of 14.6 ± 3 µm and 7.2 ± 2 µm for HEC-1A and EMO respectively) and the HTS-induced Ca^2+^-increase were significantly different compared to EMO (mean ΔCa^2+^  = 53 ± 6 nM for HEC-1A) (Fig. 5c–e). In contrast, calcium responses to Yoda1 (mean ΔCa^2+^  = 374 ± 39 nM) were comparable to EMO whereas the responses to trypsin stimulation were three times higher in HEC-1A cells (mean ΔCa^2+^  = 1325 ± 50 nM) (Fig. 5g).

In conclusion, these data indicate that EMO in general retain a comparable endometrial epithelial ion channel expression pattern as observed in hEEC, even over longer periods of culture. Moreover, the fingerprint of EMO for mechanosensitive ion channels is more comparable to the primary culture hEEC than the endometrial cancer HEC-1A cell line. However, both cellular models of endometrial epithelial cells showed reduced expression of *PIEZO1* compared to hEEC.

## Discussion

Studies assessing the underlying molecular mechanisms behind the embryo-uterine interplay are very scarce and molecular candidates involved in the early communication still need to be fully elucidated and characterized. Ion channels are potential candidates to govern signals and are emerging more often as important players in the field of reproductive medicine^42,43^. However, the fingerprint of ion channels in endometrial epithelial cells is very incomplete.

As the communication between blastocyst and epithelium can be both chemical and mechanical, the expression of mechanosensitive ion channels in endometrial epithelial cells of mouse and human was investigated. Importantly, mechanical stimulation of EEC resulted in robust Ca^2+^ influxes, and transient increases in current densities, that were not detected in the absence of extracellular Ca^2+^ and could be blocked in the presence of GsMTx4, an inhibitor of mechanosensitive channels. These results indicate for the first time the functional expression of one (or more) mechanosensitive ion channel(s) in endometrial epithelial cells of mouse and human. A first possible candidate is the PIEZO1 channel^13^ since high mRNA-expression levels were detected in hEEC and in endometrial biopsies during different stages of the menstrual cycle. Similar expression results were detected in mouse that were supported by *in situ* hybridisation experiments showing positive staining for *Piezo1* RNA in EEC of the mouse uterine horn. Importantly, stimulation of EEC by the chemical agonist Yoda1 evoked robust Ca^2+^ influxes and resulted in an increase in current amplitudes in whole-cell patch clamp experiments. Corresponding results using mechanical and chemical activation, were obtained in HEK-293T cells overexpressing mouse PIEZO1, as previously demonstrated by several groups^13,30,44^, suggesting the functional expression of PIEZO1 as an endogenously expressed mechanosensitive ion channel in EEC of mouse and human. As homologous deletion of PIEZO1 in mice is embryonically lethal and *Piezo1*^*−/−*^ pups die at mid gestation (E9.5–E11.5)^45^, the possibilities to gain further insights in the physiological role of PIEZO1 in the implantation process are limited and further research is necessary to underpin the role of PIEZO1 in embryo implantation.

Next, the expression of mechanosensitive K^+^ channels was investigated by RT-qPCR. However, the expression levels of *KCNK2* and *KCNK4* were below the detection limit in mouse and human cells, excluding them as a candidate mechanosensors in EEC. Moreover, additive experiments revealed mRNA expression of the mechanosensitive *TRPV1/V2/V4*, and *TRPC1/C4/C6* in hEEC. However, Ca^2+^-microfluorimetric experiments showed no Ca^2+^ influx after stimulation with specific TRP channel agonists, indicating the lack of functional expression of these polymodal TRP channels. However, RT-qPCR studies and Ca^2+^ imaging experiments did indicate the functional expression of TRPM7, illustrating the correlation between RNA expression levels and functional protein expression in hEEC. The epithelial character of the endometrial cells was confirmed by the high mRNA expression of *ENaC* and the robust Ca^2+^ responses to trypsin stimulation. The trypsin-induced Ca^2+^ increase was assigned to the opening of voltage dependent calcium channels by an ENaC-induced membrane depolarization. This is in accordance with earlier studies describing a role for ENaC in triggering prostaglandin E_2_ production and release from the endometrial epithelium, required for embryo implantation^12^. Previous reports have described a role for ENaC in electrolyte and water reabsorption during the peri-implantation period in mice^46^ and its function in the disappearance of uterine fluid, or the closure of the uterine lumen^47^. Although ENaC is described in some studies as a channel that can be activated by mechanical force^10,11^, several controversial results have been published on the mechanosensitivity of the channel^48^. Overall, these results provide strong evidence for the functional expression of PIEZO1 as mechanosensitive ion channel in the endometrial epithelial cells of human and mouse.

In addition, RT-qPCR experiments revealed mRNA expression of *TRPM4* and *TRPM7* in hEEC. Overall, the expression pattern of TRP channels in EEC is very limited and in strong contrast to the fingerprint that was observed in human endometrial stromal cells^38^. These results are in line with the distinct expression pattern of TRP channels described in endometrial epithelial and stromal cells in mouse. In mice, the overall expression of TRP channels in endometrial epithelial cells was very low, except for *Trpv4*, *Trpv6*, *Trpm4* and *Trpm6* that were significantly higher in epithelial cells as compared to stromal cells^36^. Remarkably, the gene expression of *TRPV4* and *TRPM6* in hEEC is situated around the detection level and thus lower as compared to the mouse expression pattern. These data are supported by the fact that no correlation could be detected in TRP channel expression pattern between mouse and human endometrial epithelial cells (Spearman correlation = 0.10; p = 0.746) (Supplementary Fig. S10). Stimulation with a TRPV4 agonist did not induce any Ca^2+^ influx, indicating no functional expression of TRPV4 in human. This outcome is in contrast with mouse EEC where stimulation by GSK induced robust Ca^2+^ influxes which could be blocked by the TRPV4 inhibitor HC067047^36^. This discrepancy between mouse and human could be explained as a species difference. Alternatively, there could be a difference in origin of the epithelial cells, since it is currently unclear whether luminal epithelial cells share a similar expression pattern compared to the glandular epithelial cells. Most probably, the primary culture of mouse EEC originates from the luminal epithelial, whereas the origin of human EEC is unclear. Or else, it is possible that a difference in culture conditions between mouse (1–2 days in culture) and human EEC (2–4 days) induces changes in the expression levels. In addition, *TRPV6* gene expression levels were significant in both mouse and human epithelial cells as visualized by *in situ* hybridization studies in mouse, and could suggest an important role for TRPV6 in the embryo implantation process. Indeed, female mice lacking functional TRPV6 are sub-fertile, showed an increased latency to pregnancy and displayed a smaller litter size^49^. Nonetheless, *TRPV6* expression was lower in human cells compared to mouse. Since *TRPV6* expression in the Ishikawa cell line is upregulated by E2, the increased *Trpv6* levels in mouse EEC could be explained by the difference in isolation protocol in which the animals are treated by high doses of E2^50^.

Unravelling the paracrine signalling between the blastocyst and the endometrium *in vitro* is often restricted as obtaining, isolating and culturing hEEC has proven challenging. Alternatively, many groups resort to endometrial epithelial cell lines, such as HEC-1A, Ishikawa cells, ECC-1, RL95-2, and others to overcome this problem. Most of these cell lines are established from endometrial adenocarcinomas several decades ago and have distinct properties in terms of adhesiveness and the presence of hormones, receptors and adhesion molecules^41,51,52^. Moreover, aside from their cancer profile, it has been shown that the long-term culture and use of these cell lines can result in loss of integrity and genetic changes^51,53^. Recently, endometrial epithelial organoids (EMO) have been proposed as an alternative research tool in implantation studies^25,40^. The EMO are derived from endometrial biopsies, but exhibit the advantage to be established from limited amounts of starting material, expanded into high cell yields and kept in culture for long periods of time. The organoids phenocopy physiological responses of the endometrial epithelium to hormones and can replicate the menstrual cycle under hormonal treatment^25^. In addition, when seeded on standard culture plates, EMO-derived cells establish a 2D monolayer that resembles the morphology of hEEC. Together, these characteristics make organoids more appealing to use in *in vitro* studies. This study has evaluated the expression pattern of ion channels in EMO and compared this fingerprint to primary hEEC. Overall, these results showed corresponding functional expression patterns of mechanosensitive and TRP channels between hEEC and EMO, although there may be a tendency of lower expression levels in EMO. A plausible explanation could be the difference in culture conditions: whereas hEEC are cultured in the presence of serum, the organoids are expanded in serum-free medium. Moreover, the organoids are cultured in the presence of estradiol which makes them representative of the proliferative phase of the menstrual cycle^25^. Alternatively, the potential significance in expression pattern can be due to the fact that the biopsies are isolated from patients undergoing fertility treatment and might have an altered expression pattern. In addition, the origin of the epithelial cells in EMO is not known and may differ from the origin of the hEEC. Nevertheless, the TRP channel expression pattern of hEEC showed a positive correlation compared to EMO (Spearman correlation = 0.66; p < 0.05). Intriguingly, expression of the *PIEZO1* channel was not detected in 3D organoids, but mechanical stimulation of the cell membrane by poking or application of a hypotonic solution and ligand stimulation resulted in Ca^2+^ influxes in the organoid-derived 2D cultures, suggesting that expression was restored. Indeed, RT-qPCR results on 2D and 3D cultures of organoids showed increased *PIEZO1* RNA expression upon 2D culture conditions. The effect of 3D culture on mechanical transduction and signalling has already been illustrated^54^ and therefore, the altered cell conformation may have induced an upregulation of PIEZO1, independent of FBS supplementation to the culture medium.

Further, the effect of long-term expandability of EMO was investigated by evaluating the expression of a selected panel of ion channels in early (P2) and late passage long-term EMO culture (P8). Overall, no significant differences were noticed between early and late passages for gene- and functional expression, supporting the stable character of the EMO.

Finally, the expression pattern of hEEC and EMO for mechanosensitive ion channels was further compared to that of the endometrial cancer cell line HEC-1A. In general, these results showed a very low expression of ion channels in HEC-1A cells, even the expression of the epithelial marker *TRPV6* was below the detection level. Moreover, no correlation was observed between the expression pattern of ion channels between hEEC and HEC-1A. Mechanical and ligand stimulation of HEC-1A cells induced influxes in intracellular Ca^2+^ concentration, indicating the presence of functional PIEZO1. However, the sensitivity of HEC-1A cells towards mechanical stimulation was significantly reduced compared to EMO as illustrated by the increased indentation necessary to evoke a Ca^2+^ influx and the reduced response to application of a HTS. Moreover, HEC-1A cells displayed significantly larger Ca^2+^ influxes towards trypsin stimulation compared to hEEC and EMO (Fig. 5g). This modified response towards trypsin stimulation can be explained by an altered expression pattern of other proteins involved in the influx or release of Ca^2+^. Since trypsin is a non-selective agonist, it cannot be excluded that this stimulus acts on different proteins involved in inducing an increase in Ca^2+^ concentration. An altered gene-expression could also explain the carcinogenic character of the HEC-1A cells and their ability to survive long-term culture. Overall, these data argument that EMO are a more representative *in vitro* model to study mechanosensitive ion channels in implantation compared to HEC-1A cells that are derived from an endometrial adenocarcinoma obtained from a patient of non-reproductive age^55^. In contrast, primary EMO are obtained from control tissue at reproductive age and closely replicate the endometrial epithelium in terms of expression of specific markers, mucus production, responsiveness to regulatory hormones and simulation of the human menstrual cycle^25^.

Altogether, these experiments validate for endometrial organoids as a representative model for primary EEC and provide further support for the use of EMO as an *in vitro* model to study interactions between the blastocyst and the endometrial epithelium.

In conclusion, this research concentrates on the expression of mechanosensitive ion channels in EEC that might be crucial in the embryo-uterine crosstalk. Our results provide strong evidence for the functional expression of PIEZO1 in EEC of mouse and human, suggesting a potential role as signal transducer in embryo implantation. Additionally, we showed a specific expression pattern of TRP channels in hEEC, which is distinct from the earlier described expression in endometrial stromal cells. Finally, our results validate endometrial organoids as a solid *in vitro* implantation model to examine the role of mechanosensitive ion channels in embryo implantation.

## Methods

### Sample collection

#### Ethical approval

All samples were collected at the Leuven University Fertility Centre with the approval of the Ethical Committee of the University Hospital Gasthuisberg, Leuven, Belgium (S54776, S60959 and S59006) and after written informed consent of the patient. By signing the informed consent patients agreed to publish obtained results. All research was performed accordance the approved guidelines and regulations.

#### Collection of human endometrial biopsies

Endometrial biopsies were obtained from patients of reproductive age who underwent the procedure as part of the diagnostic examination in their fertility treatment. Endometrial biopsies were taken using a sterile pipelle canulla or Novak curette.

For the isolation of primary EEC, biopsies were obtained from patients who were in the luteal phase of their menstrual cycle free of any hormonal medication prior to the procedure.

### Cell culture

#### Isolation of primary human endometrial epithelial cells

The protocol used is based on previously described methods^56–58^.

Immediately after the collection of the endometrial biopsy, the tissue was rinsed with phosphate buffered saline (PBS) to remove all blood and mucus. The biopsy was minced into 1 mm^2^ pieces and incubated in phenol-red free, low glucose DMEM supplemented with 1 mg/ml collagenase type I (Sigma-Aldrich, Belgium) for 60 min at 37 °C on a shaker, or overnight at 4 °C. After incubation, the resultant cell mixture was shaken thoroughly and poured through a 20 µm cell strainer. The filtrate containing the endometrial stromal cells (hESC) was centrifuged for 5 min at 720 × *g*, resuspended in growth medium containing DMEM/F12 supplemented with 10% fetal bovine serum (FBS), 0.2% gentamycin and 0.2% amphotericin B, and seeded in the appropriate wells, whereas the EEC were retained by the strainer. The epithelial fraction was collected by backwashing the cell strainer with growth medium consisting of phenol-red free, low glucose DMEM, 10% FBS, 20% MCDB-105, 5 µg/ml insulin, 0.2% gentamycin and 0.2% amphotericin B. The flow-through was mechanically disrupted by gently pipetting the solution up and down, collected in a T75 flask and incubated for a minimum of 30 min at 37 °C in 5% CO_2_. After incubation, the cells were collected, centrifuged at 720 × *g*, resuspended in growth medium and seeded in the appropriate cell culture plates for further experiments. Cells were kept at 37 °C in 5% CO_2_ and the medium was changed every 2 days. All experiments were performed on EEC at P0.

#### Isolation of primary mouse endometrial epithelial cells

Primary mEEC and mESC cells were isolated as previously described^32^.The use of mice for these experiments was approved by the ethical committee for animal experiments and welfare of the KU Leuven (p102/2017). All experiments were performed according to the approved guidelines and regulations.

#### Human endometrial organoids

Human endometrial organoids (EMO) were generated as previously described by Boretto *et al*.^25^. Endometrial biopsies were obtained from hormonally non-treated patients undergoing laparoscopy for benign gynecological conditions. All experiments were performed with EMO of early passage (P1-3) or late passage (P8).

#### Culture of HEC-1A and HEK-293T cells

The HEC-1A endometrial adenocarcinoma cell line was kindly provided by F. Vilella and C. Simón and cultured using growth medium consisting of McCoys 5A modified medium, 10% FBS, 0.2% gentamycin and 0.2% amphotericin B. HEK-293T cells were cultured as previously described^59^, and transiently transfected with 2 µg of murine *Piezo1* cDNA construct (kindly provided by A. Patapoutian) using Mirus TranIT-293 transfection reagent (Mirus Corporation, USA) 72 hrs before measurements. Transfected cells were visualized by green fluorescence protein (GFP) expression. GFP-negative cells from the same batch were used as controls.

### Immunocytochemistry

Human EEC were seeded in a 12-well plate on collagen-coated (Sigma-Aldrich, Belgium) coverslips (Karl Hecht, Germany). The growth medium was removed and cells were fixed with a 1:1 mixture of EtOH and 4% paraformaldehyde for 1 min, permeabilized with 0.25% Triton X-100 for 10 min, and blocked with 3% normal goat serum for 15 min. The primary monoclonal antibody mouse anti-human MMP-2 (0.5 µg/ml; 1/200) (Abcam, United Kingdom) or polyclonal rabbit anti-human MMP-7 (0.5 µg/ml; 1/40) (Abcam) was incubated overnight at 4 °C. The cells were further incubated for 1 h with horseradish peroxidase-labelled secondary goat anti-rabbit or goat anti-mouse antibodies (1/100, EnVision system) (Agilent, Belgium). The substrate was stained with diaminobenzidine (DAB) (Sigma Aldrich, Belgium) and counterstained with Maeyer Haematoxyline.

### RNAscope *in situ* hybridization assay

The RNAscope Multiplex Fluorescent Reagent Kit (Advanced Cell Diagnostics, US) was used for the *in situ* hybridization (ISH) of *Piezo1* in mouse uterus, bladder and trigeminal neurons. The ISH assay was performed according to the manufacturers’ guidelines for formalin-fixed, paraffin-embedded samples. In brief, tissue samples were pre-treated via the application of RNAscope hydrogen peroxide and Protease IV. Murine *Piezo1* or *Trpv6* specific probe (Advanced Cell Diagnostics, US) was applied for 2 h at 40 °C in a HybEZ oven. Samples were washed, and signal amplification steps were carried out. The signal was detected using fluorescently labelled probes and sections were incubated with DAPI.

### RNA extraction and RT-qPCR

Total RNA from hEEC and endometrial cell lines was isolated, after 2–4 days of culture, using the RNeasy Mini kit (Qiagen, The Netherlands). For the EMO, the RNeasy Micro kit (Qiagen, The Netherlands) was used. RNA was isolated according to the manufacturers’ guidelines. RNA concentration and quality were assessed via the Nanodrop method (Isogen Life Science, Belgium) and Experion RNA Analysis kit (Bio-Rad, Belgium). cDNA was generated from 1 µg of RNA using the First-Strand cDNA Synthesis Kit (GE Healthcare, Belgium). RT-qPCR was performed on triplicate cDNA samples using specific TaqMan gene expression assays (Supplementary Table I) (Life Technologies, Belgium) in the StepOne PCR system (Applied Biosystems, Belgium). Hypoxanthine Phosphoribosyltransferase 1 (*HPRT1*) and Phosphoglycerate Kinase 1 (*PGK1*) were used as endogenous controls. Data is represented as mean ± SEM of 2^(−ΔCt)^ for which ΔCt = Ct_gene of interest_ − Ct_geometric mean of HPRT1 and PGK1_.

### Functional measurements

#### Pharmacology

Functional PIEZO1 and ENaC activity was evaluated using 5 µM 2-[5-[[(2,6-Dichlorophenyl)methyl]thio]-1,3,4-thiadiazol-2-yl]pyrazine (Yoda1) (Interchim, France) and 2 µg/ml trypsin respectively. *Grammostola spatulata* mechanotoxin 4 (GsMTx4, 20 µM) (Alomone Labs, Israel) was used to challenge mechanically induced responses. TRP channel responses were measured using 50 µM *Δ*^9^*-*tetrahydrocannabinol (THC) for TRPV2, 10 nM GSK1016790A (GSK) (Sigma-Aldrich, Belgium) for TRPV4, 250 nM (−) Englerin A (EA) (Phytolabs, Germany) for TRPC1/4, 100 µM 1-oleoyl-2-acetyl-glycerol (OAG) (Calbiochem, The Netherlands) for TRPC6, and 200 µM mibefradil (Mib) (Sigma-Aldrich, Belgium) for TRPM7. HC-067047 (HC, 100 nM) (Sigma-Aldrich, Belgium) was used to challenge TRPV4-induced responses. 2 µM ionomycin was applied as positive control at the end of each experiment. All stock solutions were prepared in either DMSO, Milli Q water or EtOH.

#### Calcium imaging

The measurement of intracellular Ca^2+^ was performed as previously described^36^. Absolute calcium concentrations were calculated from the ratio of the fluorescence signals at both wavelengths (F340/F380) after correction for the individual background fluorescence signals, using the Grynkiewicz equation^60^:

$$[{{\rm{Ca}}}^{2+}]={{\rm{K}}}_{{\rm{eff}}}\,\frac{{\rm{R}}-{{\rm{R}}}_{0}}{{{\rm{R}}}_{1}-{\rm{R}}}$$

where the calibration constants R_0_, R_1_ and K_eff_ were determined as followed: R_0_ defines the ratio in Ca^2+^ free medium supplemented with 10 mM EGTA, whereas R_1_ comprises the ratio in high Ca^2+^ medium (10 mM). K_eff_, the effective binding constant, includes R_0_, R_1_, the dissociation constant of indicator dye K_D_, and the isocoefficient α, according to the following equation:

$${{\rm{K}}}_{{\rm{eff}}}={{\rm{K}}}_{{\rm{D}}}\,\frac{{{\rm{R}}}_{1}+{\rm{\alpha }}}{{{\rm{R}}}_{0}+{\rm{\alpha }}}$$

The K_D_ of Fura-2 and the isocoefficient α were assumed as described by Zhou and Neher^61^.

Cells were considered responders if the amplitude of the rise in intracellular calcium during agonist application exceeded 100 nM and when the highest value of the derivative of the calcium trace during the application of an activator exceeded at least 3 times the standard deviation of the derivative during basal conditions. Calcium amplitudes were calculated as the difference between the maximum calcium and basal calcium of responding cells during the application of an activator. Only cells that responded to the positive control, ionomycin, at the end of the experiment were taken into account. For all measurements, the following bath solution was used (in mM): 150 NaCl, 2 CaCl_2_, 1 MgCl_2,_ 10 D-glucose and 10 HEPES (pH 7.4 with NaOH). For measuring the swelling-activated calcium influx, an isotonic solution was used containing (in mM): 105 NaCl, 6 CsCl_2_, 5 CaCl_2_, 1 MgCl_2,_ 10 D-glucose, 10 HEPES and 90 D-mannitol, pH 7.4 with NaOH (320 milliosmolar). Cell swelling was induced by omitting mannitol from this solution, and reducing NaCl to 80 mM (giving 210 milliosmolar). The measurements under calcium-free conditions were performed with similar solutions in which 5 mM CaCl_2_ was omitted and replaced with 5 mM EDTA. All experiments were performed in triplicate (n ≥ 3) on at least 80 cells.

#### Mechanical stimulation of cells

Mechanical stimulation was applied via a glass probe using the E-625 piezo controller (Physik Instrumente, Germany) with computer interface and interpreter. Calcium signals were evaluated after incubation of the cells with 2 μM Fura-2 acetoxymethyl ester for 30 min at 37 °C. The fluorescent signal was detected during alternating illumination between 340 and 380 nm on a CellˆM fluorescence microscope (Olympus, USA). The ratio of the fluorescence values at the two wavelengths (F340/F380) was determined after correction for the individual background signals. The absolute intracellular calcium values were calculated from the fluorescence ratios using the Grynkiewicz equation as described above^60^. For all measurements, the following bath solution was used (in mM): 150 NaCl, 2 CaCl_2_, 1 MgCl_2,_ 10 D-glucose and 10 HEPES (pH 7.4 with NaOH).

#### Whole-cell patch clamp

Whole-cell patch clamp recordings were measured with an EPC-10 amplifier and the PatchMasterPro Software (HEKA Elektronik, Lambrecht, Germany). Current measurements were performed at a sampling rate of 20 kHz and currents were digitally filtered at 2.9 kHz. In all measurements, 70% of the series resistance was compensated. The standard internal solution contained (in mM): 100 Asp, 45 CsCl, 10 EGTA, 10 HEPES, 1 MgCl_2_ (pH 7.2 with CsOH) and the standard extracellular solution contained (in mM): 150 NaCl, 10 HEPES, 10 Glucose, 2 CaCl_2_, 1 MgCl_2_ (pH 7.4 with NaOH). The standard patch pipette resistance was between 2 MΩ and 4 MΩ when filled with pipette solution.

### Data and statistical analysis

Ca^2+^ microfluorimetric and electrophysiological data were analyzed using home-written routines in IgorPro 6.37 (WaveMetrics, USA), and OriginPro 9 (OriginLab Corporation, USA) was further used for data display. Statistical analysis was conducted via GraphPad Prism version 7.04 (GraphPad Software, USA).

## Supplementary information

Supplementary Dataset

## Data Availability

The datasets generated during the current study are available from the corresponding author upon reasonable request.
