# Supplementary material for: Functional expression of the mechanosensitive PIEZO1 channel in primary endometrial epithelial cells and endometrial organoids
Source: Sci Rep. 2019 Feb 11;9:1779. doi: 10.1038/s41598-018-38376-8 (PMC6370865; doi:10.1038/s41598-018-38376-8)
Supplement: Supplementary file 1 — Supplementary Dataset [file 41598_2018_38376_MOESM1_ESM.pdf]

Supplementary information

Functional expression of the mechanosensitive PIEZO1 channel in primary endometrial epithelial cells and endometrial organoids

Aurélie Hennes[1,2], Katharina Held[1,2], Matteo Boretto[3], Katrien De Clercq[1,2], Charlotte Van den Eynde[1,2], Arne Vanhie[1,4], Nele Van Ranst[2], Melissa Benoit[2], Catherine Luyten[1], Karen Peeraer[1,4], Carla Tomassetti[1,4], Christel Meuleman[1,4], Thomas Voets[2], Hugo Vankelecom[3] and Joris Vriens[1,*]

Supplementary part

TABLES

Supplementary Table I. Overview of the TaqMan Gene Expression Assays (Life Technologies, ThermoFisher Scientific)

Gene	Entrez gene ID	Assay ID
**Piezo1**	9780	Hs00207230_m1
**Piezo2**	63895	Hs00926218_m1
**KCNK2**	3776	Hs01005159_m1
**KCNK4**	50801	Hs05042327_s1
**ENaC (SCNN1a)**	6337	Hs00168906_m1
**TRPA1**	8989	Hs00175798_m1
**TRPV1**	7442	Hs00218912_m1
**TRPV2**	51393	Hs00901648_m1
**TRPV4**	59341	Hs01099348_m1
**TRPV6**	55503	Hs00367960_m1
**TRPC1**	7220	Hs00608195_m1
**TRPC3**	7222	Hs00162985_m1
**TRPC4**	7223	Hs01077392_m1
**TRPC5**	7224	Hs00202960_m1
**TRPC6**	7225	Hs00988479_m1
**TRPC7**	57113	Hs00220638_m1
**TRPM1**	4308	Hs00931865_m1
**TRPM2**	7226	Hs01066091_m1
**TRPM3**	80036	Hs00257553_m1
**TRPM4**	54795	Hs00214167_m1
**TRPM5**	29850	Hs00175822_m1
**TRPM6**	140803	Hs01019356_m1
**TRPM7**	54822	Hs00559080_m1
**TRPM8**	79054	Hs01066596_m1
**HPRT1**	3251	Hs02800695_m1
**PGK1**	5230	Hs00943178_g1
**MMP-2**	4313	Hs01548727_m1
**MMP-7**	4316	Hs01042796_m1

SUPPLEMENTARY FIGURES

Supplementary Fig. S1

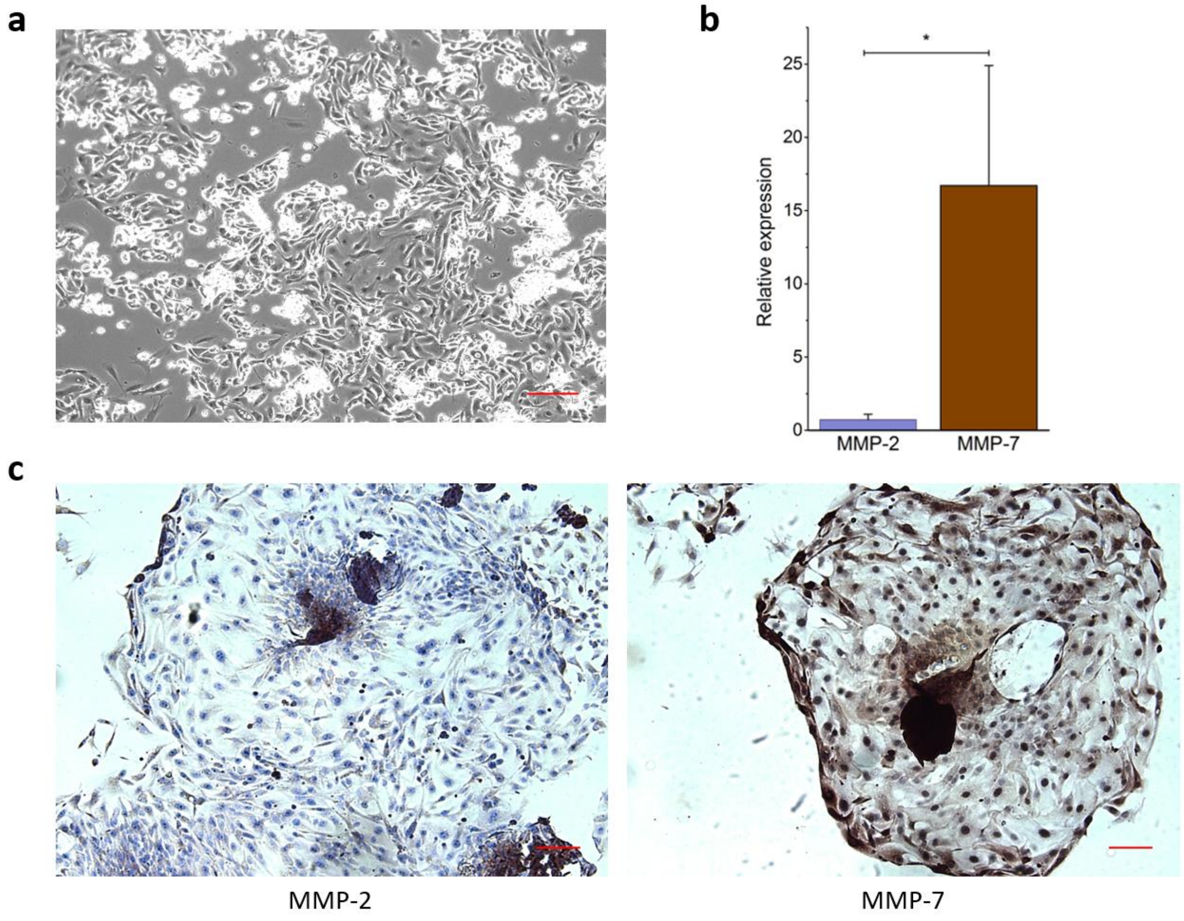

Supplementary Fig. S1. Purity of hEEC culture

(a) Representative image of hEEC culture 2 days after isolation. **(b)** mRNA expression levels of *MMP-2* and *MMP-7* in primary hEEC. mRNA levels were relatively quantified to the geometric mean of the housekeeping genes *HPRT1* and *PGK1* and represented as mean ± SEM. n = 4. * $p < 0.05$ using the non-parametric Mann-Whitney U test. **(c)** Immunocytochemical staining of MMP-2 (left) and MMP-7 (right) in cultured hEEC. Pictures were taken at 10x magnification. Scale bar = 100 µm.

Supplementary Fig. S2

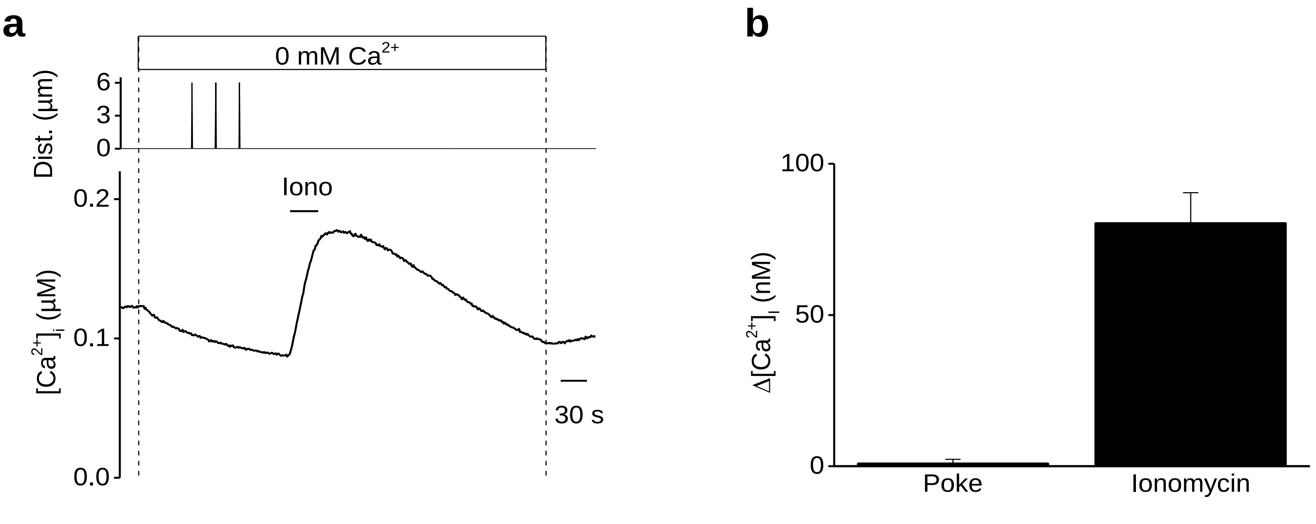

Supplementary Fig. S2. Mechanical stimulation of hEEC

(a) Mechanical stimulation of primary hEEC in 0 mM extracellular Ca^{2+}. A mechanical stimulus (6 µm) was given at the indicated time points. Ionomycin (5 µM) was applied as a positive control to deplete the intracellular Ca^{2+} stores. One representative trace is shown. N = 4 **(b)** Intracellular Ca^{2+} concentration upon mechanical stimulation (poke) or application of ionomycin as shown in (a). *Iono = ionomycin*

Supplementary Fig. S3

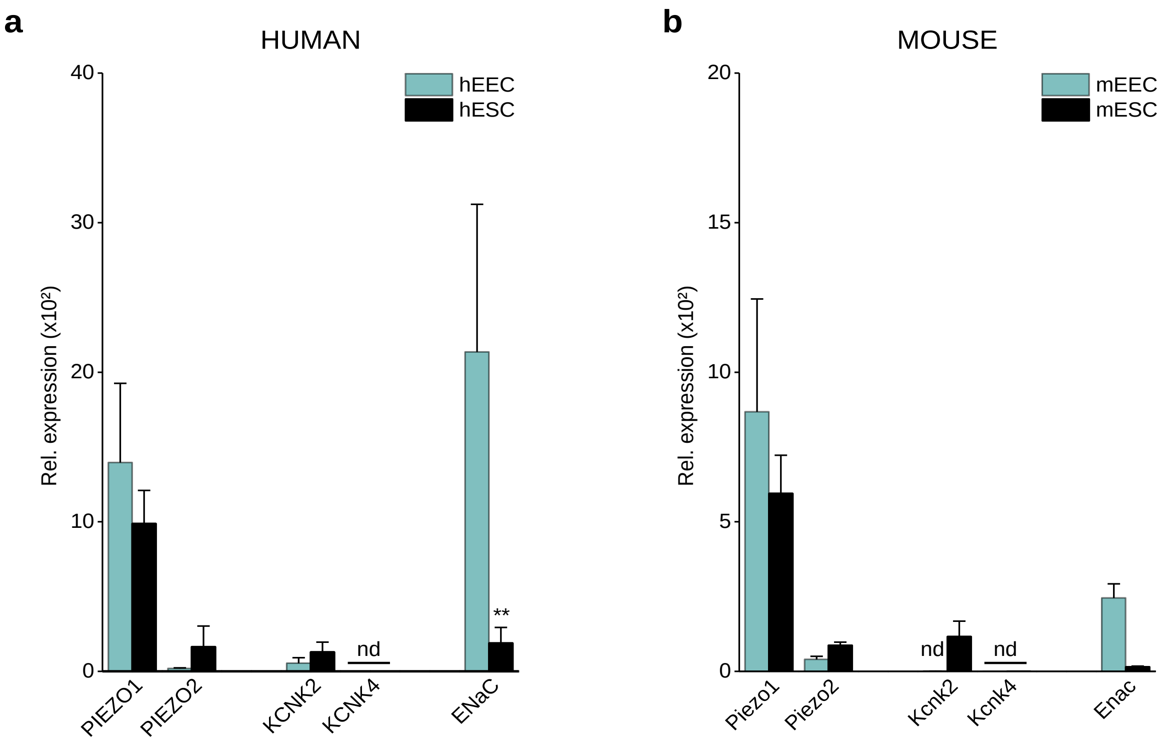

Supplementary Fig. S3. Mechanosensitive channels in human and mouse endometrial stromal cells

mRNA expression levels of mechanosensitive ion channels in primary EEC and ESC of human **(a)** and mouse **(b)**. mRNA levels were relatively quantified to the geometric mean of the housekeeping genes *HPRT1* and *PGK1* (human) or *Tbp* and *Pgk1* (mouse) and represented as mean ± SEM. ** $p < 0.01$ with Multiple t-test, corrected for multiple comparisons with the Holm-Sidak method, n = 4. Nd = not detectable.

Supplementary Fig. S4

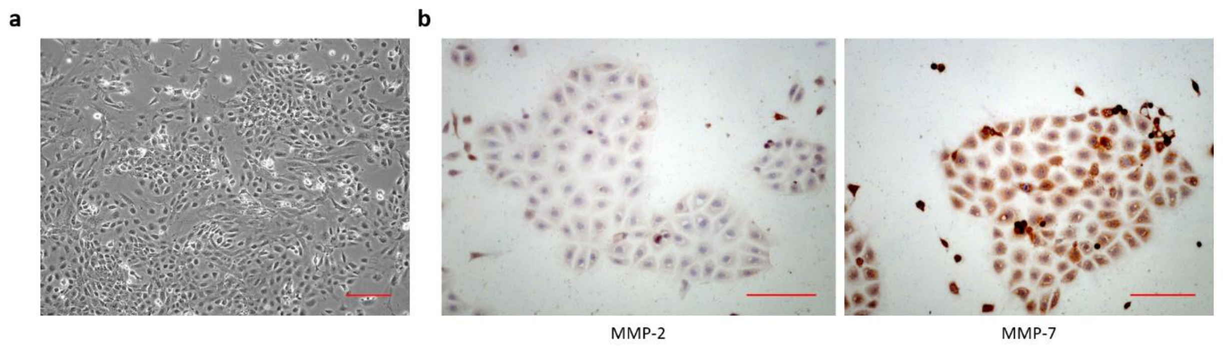

Supplementary Fig. S4. Primary culture of mouse endometrial epithelial cells

(**a**) Representative image of isolated mEEC. Picture was taken at 10x objective. Scale bar: 100 µm. **(b)** Immunostaining for the markers MMP-2 and MMP-7 in isolated mEEC. A representative picture for each marker is shown. Pictures were taken at 20x objective. Scale bar: 100 µm.

Supplementary Fig. S5

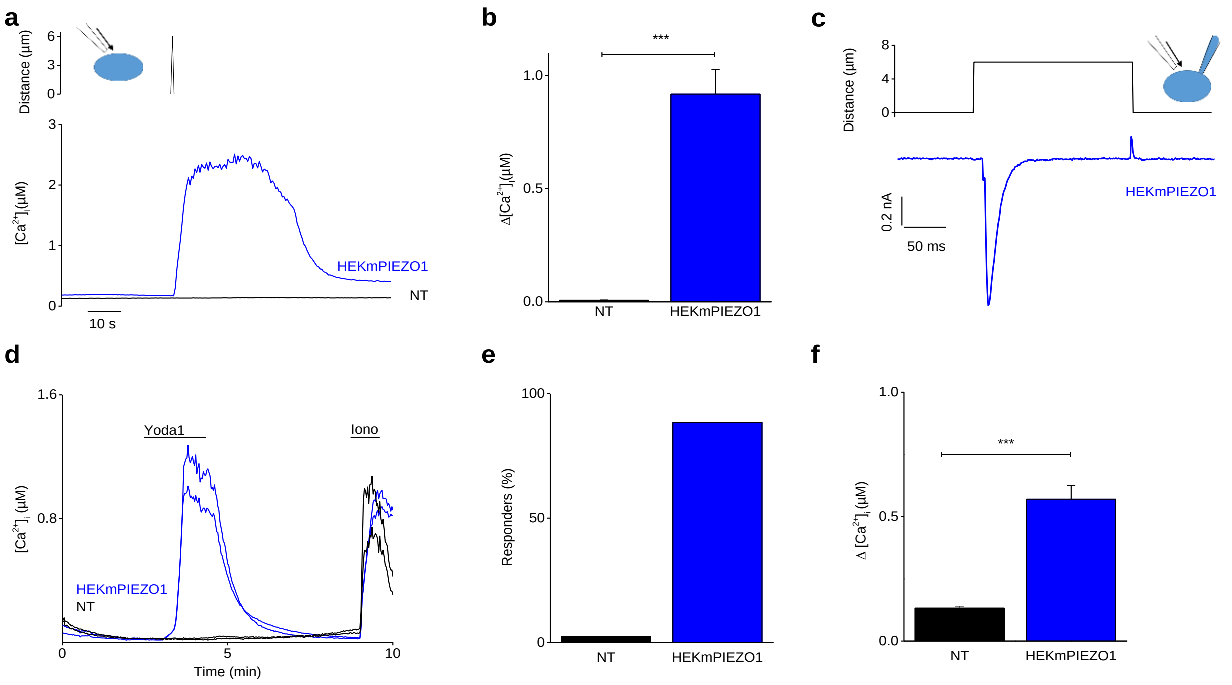

Supplementary Fig. S5. Mechanical stimulation in HEK-293T cells

(a) Time course of intracellular Ca^{2+} concentration in non-transfected HEK-293T (NT) cells (black) and HEK-293T cells overexpressing mPIEZO1 (blue). Cells were mechanically stimulated at the indicated time point (5 µm for 100 ms). One representative trace is shown. **(b)** Ca^{2+} increase upon mechanical stimulation of either non-transfected HEK293-T cells (NT), HEK-293T cells overexpressing mPIEZO1 (HEKmPIEZO1) and primary hEEC. N = 3-10. *** $p < 0.001$ using the non-parametric Mann-Whitney U test. **(c)** Representative trace of PIEZO1-dependent mechanically activated current at -60 mV in HEK-293T cell overexpressing mouse PIEZO1. **(d)** Intracellular Ca^{2+} concentration in non-transfected HEK-293T cells and HEK-293T cells overexpressing mPIEZO1. Yoda1 (5 µM) was added at the indicated time points. Ionomycin (2 µM) was applied at the end of each experiment as a positive control. Each line represents a single cell and two representative traces per condition are shown. **(e)** and **(f)** depict percentage of responders and mean Ca^{2+} influx for the experiments performed in (d). Iono = ionomycin. *** $p < 0.001$ using the non-parametric Mann-Whitney U test.

Supplementary Fig. S6

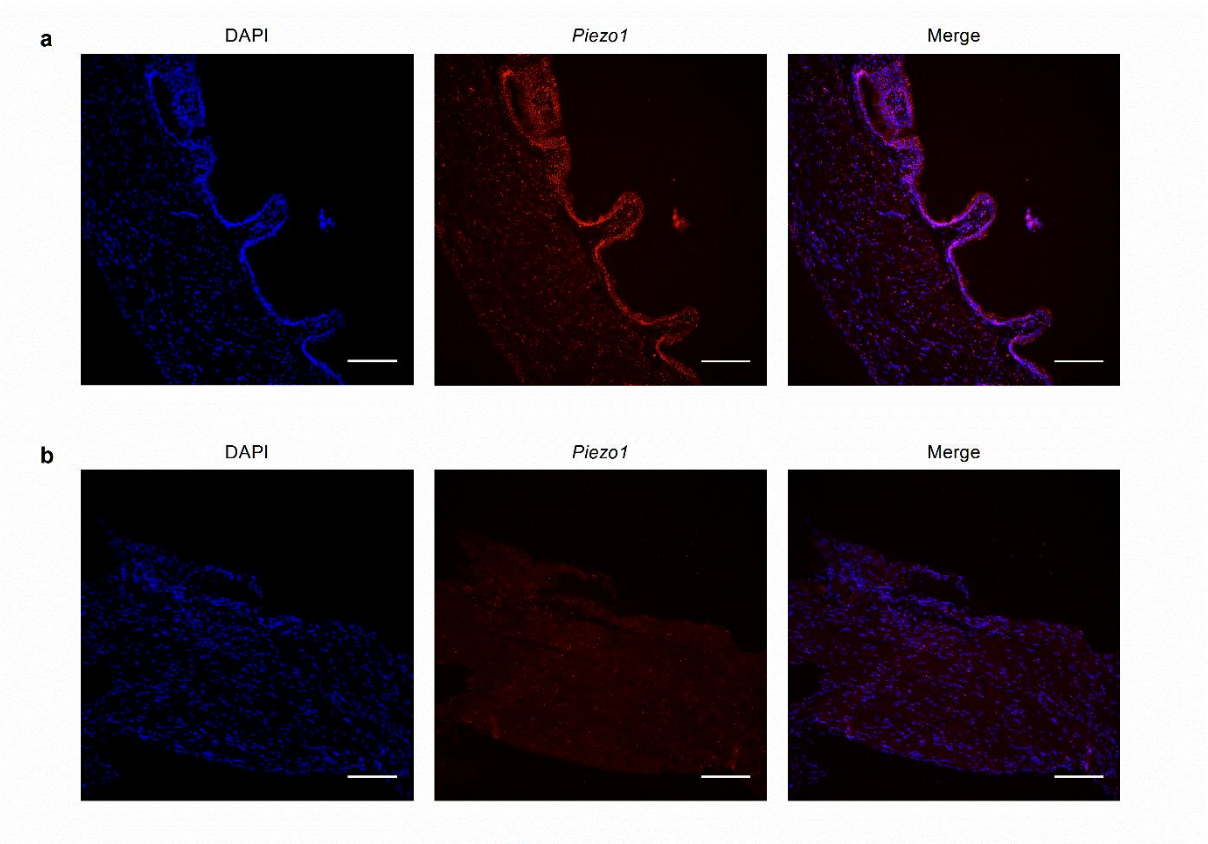

Supplementary Fig. S6. Specificity of *Piezo1* RNA *In Situ Hybridization* (ISH) probe

Representative images of mouse bladder **(a)** and mouse trigeminal neurons **(b)** stained with ISH probe specific for mouse *Piezo1*. Left panel represents DAPI image, while the middle panel shows specificity of the *Piezo1* probe. Pictures were taken at 10x objective. Scale bar: 100 µm.

Supplementary Fig. S7

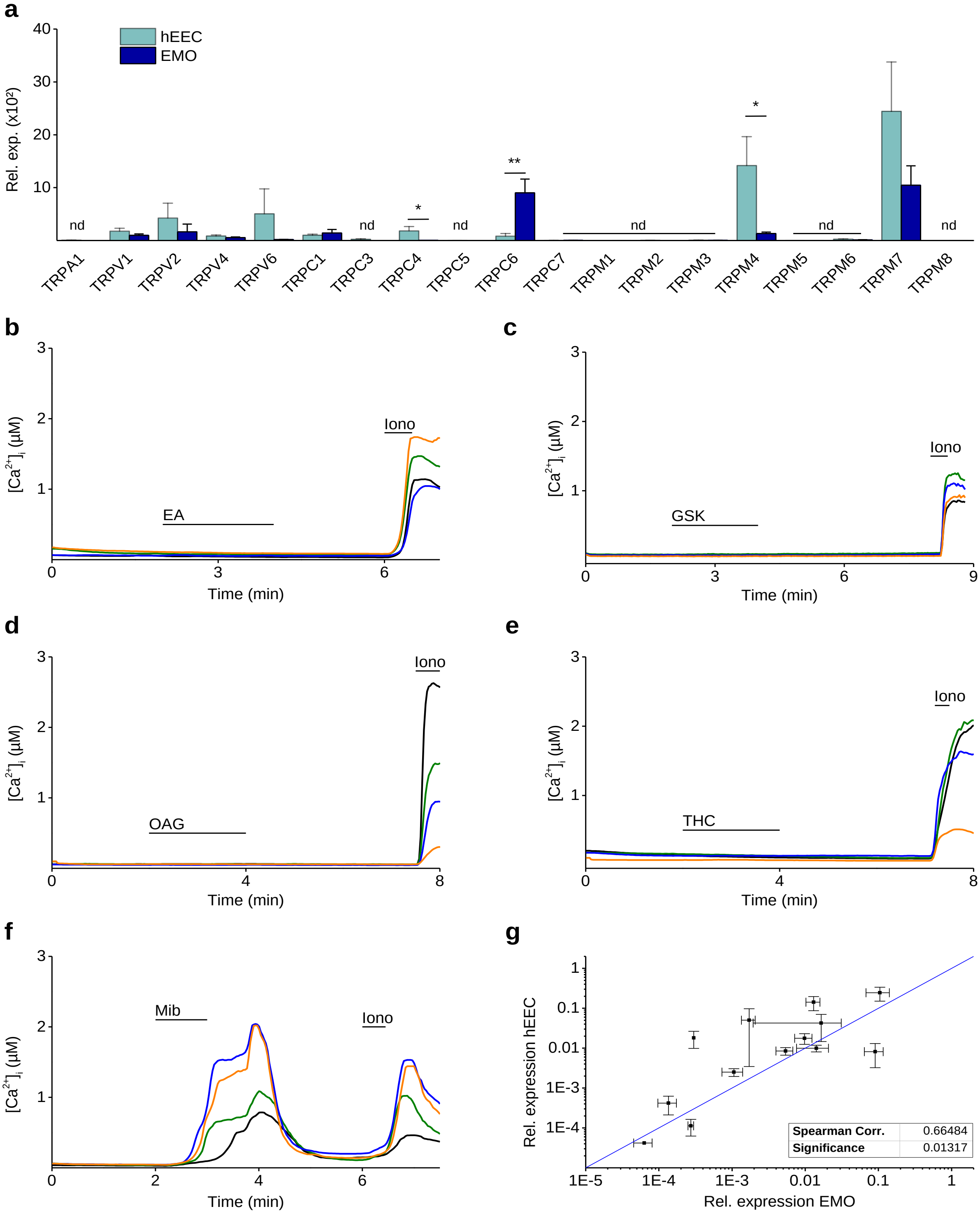

Supplementary Fig. S7. TRP channel expression and functionality in primary hEEC

(a) mRNA expression levels of TRP channels in primary hEEC (light blue bars) and early passage EMO (dark blue bars). mRNA levels were relatively quantified to the geometric mean of the housekeeping genes *HPRT1* and *PGK1* and represented as mean ± SEM. * $p < 0.05$, **

$p < 0.01$ with Multiple t-tests, corrected for multiple comparisons with the Holm-Sidak method ($n = 4$ for hEEC and $n = 3$ for EMO). Nd = not detectable. **(b - f)** Ca^{2+} microfluorimetry. Cells were stimulated with either (**b**) Englerin A (EA; 250 nM), (**c**) GSK1016790A (GSK; 10 nM), (**d**) 1-oleoyl-2-acetyl-sn-glycerol (OAG; 100 µM), (**e**) Δ^9-Tetrahydrocannabinol (THC; 50 µM), or (**f**) mibefradil (Mib; 200 µM) to induce Ca^{2+} influxes via TRPC1/C4 heteromultimers, TRPV4, TRPC6, TRPV2 or TRPM7 respectively. Ionomycin (Iono; 2 µM) was applied at the end of each experiment as a positive control. Each line represents a single cell and four representative traces are shown. N = 3 independent experiments, with a total of minimum 950 cells per condition. **(g)** Correlation between the relative mRNA expression of TRP channels in hEEC and EMO, based on the data shown in (a). The correlation was assessed using the Spearman correlation coefficient. The blue line depicts the best fitting.

Supplementary Fig. S8

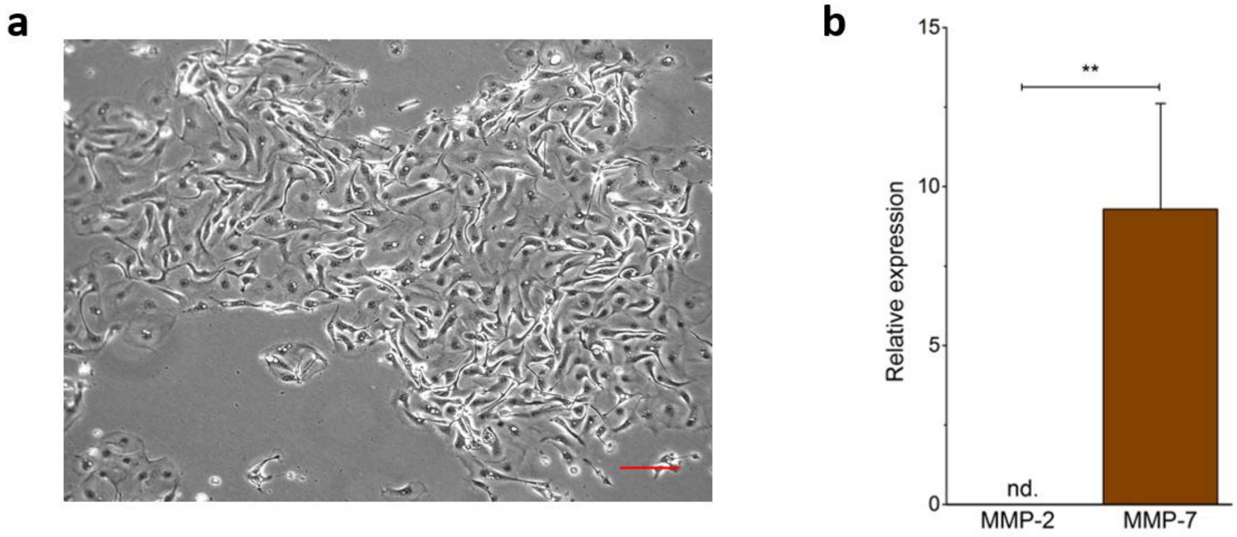

Supplementary Fig. S8. EMO-derived cell culture

(a) Representative image of cells derived from EMO when seeded in a 2D configuration. Picture was taken at a 10x objective. Scale bar: 100 µm. **(b)** mRNA expression levels of *MMP-2* and *MMP-7* in early passage (p2) EMO. mRNA levels were relatively quantified to the geometric mean of the housekeeping genes *HPRT1* and *PGK1* and represented as mean ± SEM. n = 5. ** $p < 0.01$ using the non-parametric Mann-Whitney U test.

Supplementary Fig. S9

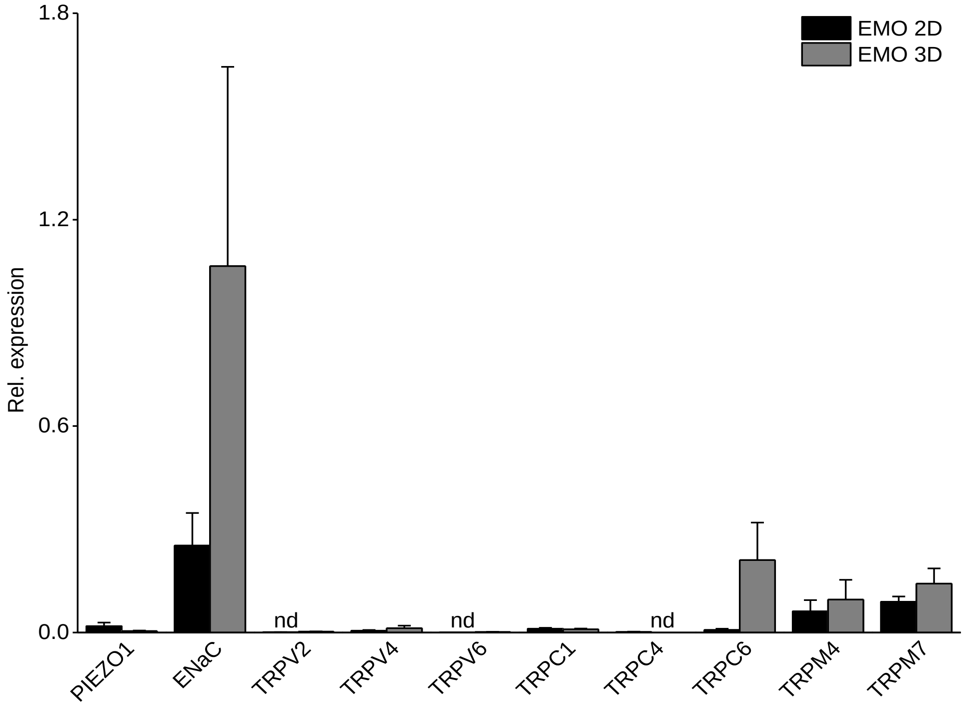

Supplementary Fig. S9. Ion channels in 2D versus 3D configuration EMO

Messenger RNA expression levels of selected ion channels in 2D and 3D cultured EMO. mRNA levels were relatively quantified to the geometric mean of the housekeeping genes *HPRT1* and *PGK1* and represented as mean ± SEM. n = 5

Supplementary Fig. S10

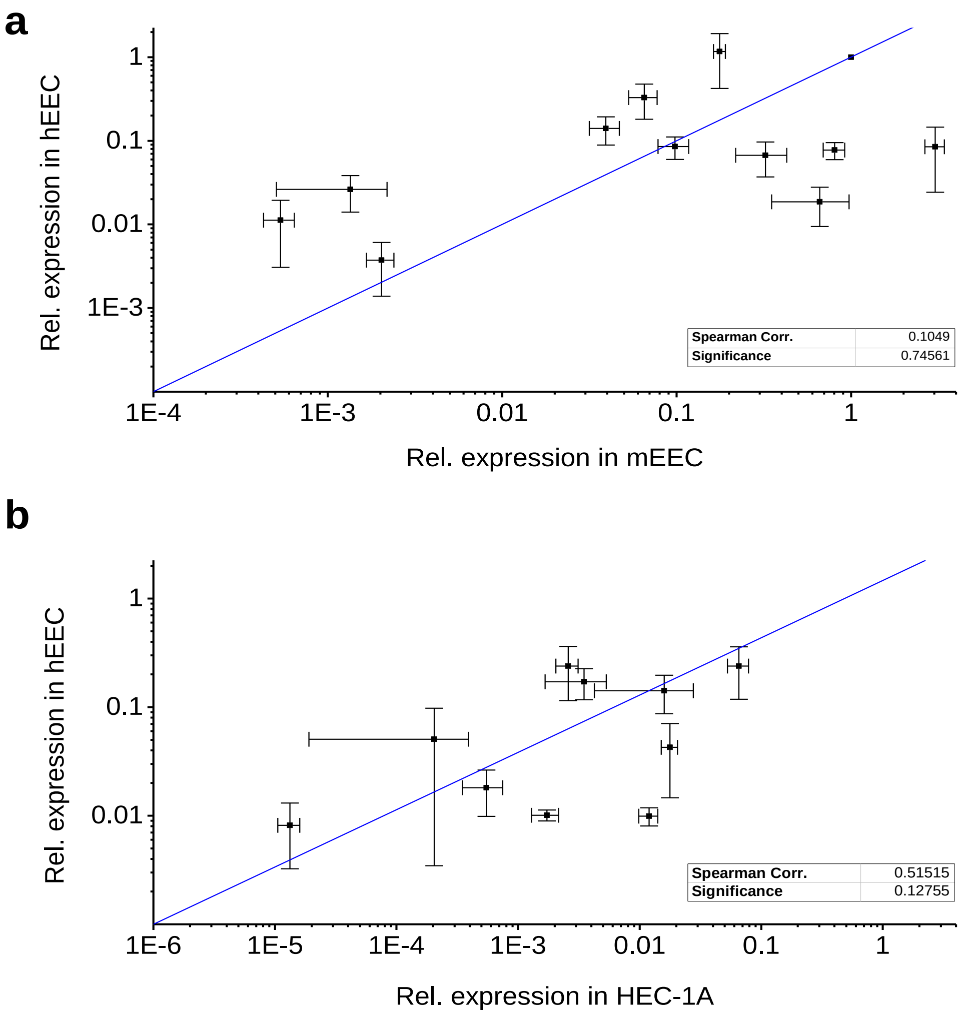

Supplementary Fig. S10. Correlation plots between hEEC / mEEC and hEEC / HEC-1A

Correlation between the mRNA expression levels of **(a)** TRP channels in hEEC (data depicted in Supplementary Fig. S7) and mEEC (data previously published by De Clercq K. *et al.* [25]). mRNA expression levels were relatively quantified against TRPM7 to cope with the differences in housekeeping genes; and **(b)** a selected panel of ion channels in hEEC versus HEC-1A. The correlation was assessed using the Spearman correlation coefficient. The blue line represents the best fitting between points.
